# Pregnancy Complicated by Portal Hypertension Secondary to Biliary Atresia

**DOI:** 10.1155/2013/421386

**Published:** 2013-12-26

**Authors:** O. E. O'Sullivan, D. Crosby, B. Byrne, C. Regan

**Affiliations:** Department of Fetomaternal Medicine, Coombe Women and Infants University Hospital, Dublin 8, Ireland

## Abstract

Biliary atresia is a rare idiopathic neonatal cholestatic disease characterized by the destruction of both the intra- and extrahepatic biliary ducts. As the disease is progressive all cases will develop portal fibrosis, cirrhosis, and portal hypertension with the sequelae of varices, jaundice, and eventually liver failure requiring a transplant. Survival rates have improved considerably with many females living well in to be childbearing age. Due to the complexity of the disease these pregnancies are considered, high risk. We report the antenatal, intrapartum, and postpartum managements of a pregnancy complicated by biliary atresia. Furthermore, we highlight the importance of a multidisciplinary team approach in optimizing obstetric care for this high risk group.

## 1. Case Presentation

DH 21-year-old primigravida presented for her booking visit at 11+2/40. Her history was notable for a background history of congenital biliary atresia (BA). She had undergone an early Kasai portoenterostomy with Roux-en-Y anastomosis in the early neonatal period. Her subsequent postoperative course was complicated by two episodes of cholangitis. By the age of two she had developed portal hypertension resulting in secondary thrombocytopenia due to hypersplenism. She had developed esophageal varices requiring annual sclerotherapy until the age of sixteen when she underwent variceal banding. Her vaccinations were up-to-date.

General findings on presentation at 11 weeks revealed icterus with scattered spider naevi. On abdominal inspection there was an upper abdominal scar from her prior surgery and on palpation the spleen was enlarged extending to below the level of the umbilicus; there was no evidence of ascites.

An ultrasound confirmed her estimated date of delivery. She was referred to the medical clinic at the hospital to provide a multidisciplinary team input to her care.

At sixteen weeks she developed an episode of cholangitis and she was treated with intravenous antibiotics for seven days. A liver ultrasound was performed. This revealed absence of intrahepatic dilatation with multiple periportal and peripancreatic varices and 14 cm splenomegaly. During the pregnancy her platelets remained low (29–55 × 10^9^ L) and hemoglobin ranged from 8.8 to 10.5 g/dL. Her transaminases were marginally elevated (AST/ALT, 46/67) and coagulation parameters were normal. Significant concern remained about her thrombocytopenia and degree of hypersplenism.

A multidisciplinary meeting was convened with hepatology, hepatobiliary surgery, and interventional radiology. Concerns for this patient included her significant splenomegaly and thrombocytopenia, risk of recurrent cholangitis, potential for massive variceal hemorrhage, and potential for preterm delivery, either spontaneous or iatrogenic. Abdominal delivery would also be compromised by large venous anastomoses in the anterior abdominal wall. A plan for management of gastrointestinal hemorrhage was outlined. Further imaging of her portal vessels was recommended as this would inform the interventional radiologists as to which was the optimum surgical intervention should bleeding develop from intraabdominal varices. The option of a splenectomy in pregnancy was excluded as this was deemed likely to cause portal vein thrombosis and would compromise any future candidacy for liver transplantation. At twenty weeks she underwent MR venography of the portal vessels. Her spleen measured 23 cm, there was evidence of extensive splenic and esophageal varices, and no lienorenal shunt was identified.

She had bimonthly antenatal visits with her obstetrical and hepatology teams. Her anatomy scan was within normal limits with a well-grown fetus on the 31st centile. Due to the complexity of the case and the potential for preterm delivery she received two doses of betamethasone at twenty-six weeks of gestation to aid lung maturation.

At 26^+3^ she presented to the maternity hospital with vaginal bleeding, suprapubic pressure, and uterine contractions for 24 hours with increasing intensity and duration. The fundal height was consistent with 26 weeks of pregnancy and cardiotocography was reassuring. Sterile speculum examination confirmed that the cervix was 5 cm dilated and fully effaced and the amniotic membranes were bulging through the cervix. A bedside ultrasound scan confirmed a breech presentation. Her platelet count was 33 × 10^9^/L. Following consultation with perioperative medicine, the hepatobiliary team, and neonatology the decision was made to perform an emergency caesarean section. It had been planned to perform her surgery in a general hospital; however, due to the acute nature of her presentation the caesarean section was undertaken in the obstetric facility, with hepatobiliary surgery and interventional radiology on standby in the general hospital. Pooled platelets were administered immediately prior to surgery. Magnesium sulphate was administered to provide fetal neuroprotection. The caesarean section was performed via a vertical subumbilical incision, with a lower segment transverse incision on the uterus. The vertical incision was utilized both to provide greater access to the abdominal cavity should bleeding occur, either from the uterus or the varices, and to avoid large venous collaterals running vertically in the abdominal wall. A live male baby was delivered by breech extraction and transferred to the pediatric team following delayed cord clamping of 45 seconds. Apgar's scores were 6^1^, 6^5^, and 7^10^; the cord blood gases were pH 7.27 (arterial) 7.29 (venous). The infant weighed 890 grams. The remainder of the surgery was uneventful. Perioperatively she received a further pooled platelet transfusion with good hemostasis and no transfusion of packed red cells was required. Her baby was transferred to intensive care and initially required CPAP. He had an uncomplicated neonatal course. Histology of the placenta was unremarkable. The patient had an uneventful postpartum course. She was advised against further pregnancies in view of her significant risks and underwent insertion of a levonorgestrel coil at three months postpartum for contraceptive purposes and she is currently awaiting liver transplantation. Despite the premature gestational age, the infant had an essentially uneventful postnatal course. He was discharged home at ten weeks of age and as a one-year-old boy he has met all his expected developmental milestones.

## 2. Discussion

Biliary atresia (BA) is a rare idiopathic neonatal cholestatic disease characterized by the destruction of both the intra- and extrahepatic biliary ducts. BA occurs most often (~90%) in isolation without the presence of other malformations but can also be a part of a syndrome. Syndromic BA can be associated with various congenital anomalies such as polysplenia or asplenia (100%), situs inversus (50%), preduodenal portal vein (60%), absence of retrohepatic inferior vena cava (40%), or cardiac anomalies (50%) [1]. BA is classified on anatomical bases, referring to the level and severity of the obstruction. The Japanese and Anglo-Saxon classification describes 3 main types. In type I, atresia is limited to the common bile duct, and the gallbladder and hepatic ducts are patent (i.e., “distal” BA). In type II, atresia affects the hepatic duct, but the proximal intrahepatic ducts are patent (i.e., “proximal” BA). Type II is subgrouped in type IIa, where a patent gallbladder and patent common bile duct are present (sometimes with a cyst in the hilum, i.e., “cystic BA”), and type IIb, where the gallbladder, the cystic duct, and common bile duct are also obliterated. In type III, there is discontinuity of not only the right and left intrahepatic hepatic ducts, but also of the entire extrahepatic biliary tree (i.e., “complete” BA). The reported incidence of BA in several European countries varies between 1/14,000 and 1/20,000 live births [2, 3].

Importantly there are two clinical manifestations of the disease: an embryonal subtype, which often presents at birth and is associated with congenital malformations, and a “perinatal” subtype, which is probably an acquired disease due to unknown etiology [4]. Death occurs within the first year of life if left untreated due to the sequelae of cirrhosis. The current treatment of biliary atresia is surgical. During the first months of life a hepatoportoenterostomy “Kasai procedure” should be performed, in order to restore the biliary flow to the intestine and lessen further damage to the liver. Almost 80% of patients become jaundice-free after Kasai [5]. If this fails and/or the disease progresses towards biliary cirrhosis and life-threatening complications, then liver transplantation is indicated. Biliary atresia represents the most frequent pediatric indication for liver transplantation. The earlier the Kasai is performed, the later a liver transplantation is usually needed, thus, placing great emphasis on systematic screening for this life-threatening pathology and subsequent early intervention [6].

The Kasai aims at restoring bile flow between the liver and the intestine, using a jejunal Roux-en-Y limb, which is anastomosed to the porta hepatis after resection of the biliary remnant. The procedure is named after the Japanese surgeon Morio Kasai, who first performed this procedure in 1959 [7].

The long-term complications of BA include cholangitis where the number of cholangitic episodes is known to negatively influence the success of the Kasai operation [8]. As the disease is progressive, all cases will develop portal fibrosis, cirrhosis, and portal hypertension with the sequelae of varices, jaundice, and eventually liver failure requiring a transplant. In some severe cases hepatopulmonary syndrome may occur with its characteristics of hypoxemia, cyanosis, and dyspnea. Despite surgical intervention intrahepatic biliary cavities either solitary or multiple occur in about 20% of cases [9]. Where liver cirrhosis develops there exists the risk of associated malignancies such as hepatocellular carcinoma, hepatoblastoma, and cholangiocarcinoma. Between 70 and 80% of patients will require liver transplantation prior to adulthood [10]. Where the Kasai procedure is unsuccessful and/or medical complications of biliary cirrhosis appear liver transplant is indicated. Most transplantations occur in the first or second year of life. The goal is to offer a normal life to these children, allowing for normal physical, intellectual, psychological, sexual, and social developments [11]. Overall survival has improved since the implementation of the Kasai operation and liver transplantation. In a UK series between 1999 and 2002, 89% of 148 patients survived four years and longer [12].

The published literature regarding pregnancy complicated by biliary atresia is sparse. One case report by Chiengthong et al. [13] from Thailand reported the case of a 17-year-old patient, who underwent Kasai procedure at 3/12 of age. She has subsequently developed portal hypertension associated with abnormal liver function, esophageal varices, and hypersplenism. At 32 weeks of gestation she presented with hematemesis and thrombocytopenia. She was stabilized using a somatostatin analogue, fluid and blood component replacements, and other supportive therapies. Once stable at 33 weeks she underwent a lower segment caesarean section with no resultant complications. She made a good postoperative recovery. The authors conclude that while pregnancy is not contraindicated it should be avoided in this group of patients. Furthermore, where pregnancy occurs intensive prenatal care is required. McMichens et al. [14] discussed the case of a woman with a pregnancy complicated by portal hypertension, bleeding esophageal varices, and hypersplenism secondary to biliary atresia who delivered a healthy infant at 36 weeks of gestation.

Kuroda et al. [15] in a retrospective review of 52 cases of BA advised that social activities and pregnancy should be managed individually according to the risk assessment for potential hepatic failure even in stable adult BA patients. Sasaki et al. [16] reviewed 169 patients treated for BA over a 36-year period, nine of whom experienced pregnancy. In the series prepregnancy no patient had jaundice and three patients had portal hypertension; these women all required special treatment. In total the nine women experienced 14 pregnancies and 11 deliveries (2xTOP, 1xIUD@35/40). All children are healthy without liver dysfunction or congenital anomalies. The postpartum complication rates increased when BA was associated with an episode of cholangitis, portal hypertension, or both. When associated with portal hypertension there was a 30% incidence of gastrointestinal bleeding. Note the intrauterine death was associated with portal hypertension. They conclude that pregnancy and delivery are not to be avoided but managed on an individual basis.

Within the literature there is no indication as to the safest mode of delivery for both mother and infant. This is due in part to the complex nature of the pregnancy and the potential for antenatal complications, which are not consistent with the disease and across patients. In our case the decision for a caesarean section was made at a multidisciplinary meeting prior to the onset of labour. Furthermore, due the gestational age and presentation of the infant (breech) a caesarean section would be indicated as the safest mode of delivery for the infant irrespective of the maternal complex medical history [17].

## 3. Conclusion

Maternal congenital anomalies complicating pregnancy are best managed in a tertiary level facility with access to surgical, radiological, perioperative, and medical specialists. Pregnancy in women after Kasai procedure is seldom encountered and infrequently reported and presents a unique set of challenges to the maternal medicine specialist. In this case diagnostic imaging and the access to interventional radiology should complications arise assisted the plan of care, as the risk of hemorrhage was significant. We believe that our case highlights the importance of multidisciplinary approach when managing complex conditions during pregnancy.
